# Behçet disease: evaluation of clinical manifestations in Turkish children

**DOI:** 10.1186/1546-0096-9-S1-P19

**Published:** 2011-09-14

**Authors:** Erkan Demirkaya, Celal Saglam, Turker Turker, Sevcan Bakkaloglu, Balahan B Makay, Bora Gulhan, Banu Acar, Nuray A Ayaz, İsmail Dursun, Adem Polat, Cetin Kocabiyik, Faysal Gok, Erbil Unsal, Ozgur Kasapcapur, Seza Özen

**Affiliations:** 1 Gülhane Military Medical Academy, Pediatric Nephrology and Rheumatology Unit, Ankara, Turkey; 2 Gulhane Military Medical Faculty, Department of Public Health, Division of Epidemiology, Ankara, Turkey; 3 Gazi University Medical School, Pediatric Nephrology and Rheumatology Unit, Ankara, Turkey; 4 Dokuz Eylul University Medical School, Pediatric Immunology and Rheumatology Unit, İzmir, Turkey; 5 Hacettepe University Medical School, Pediatric Nephrology & Rheumatology Unit, Ankara, Turkey; 6 Ministry of Health Diskapi Children's Hospital, Diskapi, Ankara, Turkey; 7 SB Istanbul Bakırköy Maternity and Childrens Education and Research Hospital, Division of Pediatric Rheumatology, Istanbul, Turkey; 8 Erciyes University Medical School, Pediatric Nephrology & Rheumatology Unit, Kayseri, Turkey; 9 Istanbul University Cerrahpasa Medical School Department of Pediatric Rheumatology, Istanbul, Turkey

## Objective

We analyzed the clinical manifestations of Pediatric Behcet disease (PED-BD) in Turkey. We also evaluated the correlation between the physician’s global assessment of disease activity (PGA) and Behçet’s Syndrome Activity Scale (BSAS) which is one of the activity indices in our cohort.

## Method

8 University hospitals in Turkey enrolled children with a clinical diagnosis of PED-BD. We examined chronologically the onset of individual symptoms in each patient. As an activity index we used BSAS which have been developed to assess the activity specifically for BD in adults. Correlation between the PGA, and BSAS was determined.

## Results

In 54 patients the mean age at the first symptom was 117.50±45.20 months. BD was suspected at a mean age of 143.56±39.63 months. The mean delay between the first symptom and BD suspicion was 27.36±27.15 months. The most common manifestations were oral ulcer 96.3% (n=52), uveitis 46.3% (n=25), genital ulcer 37% (n=20), pustuler lesion 37% (n=20), erythema nodosum 24.1%(n=13) respectively. Bilateral uveitis was found in 27.8% (n=15) patients. Pathergy phenomen was positive in 37% (n=20) patients. Family history of BD was present in 38.9% (n=21) patients. HLA-B51 carrier rate was 53.7% (n=29). BSAS was assessed for the 52 patients in our cohort and a moderate correlation between the BSAS and PGA was demonstrated (r=0.305, p=0.025).

## Conclusion

Our study indicates that BSAS may be a beneficial and practical index to define the disease activity in PED-BD and that further studies with a large cohort are required.

